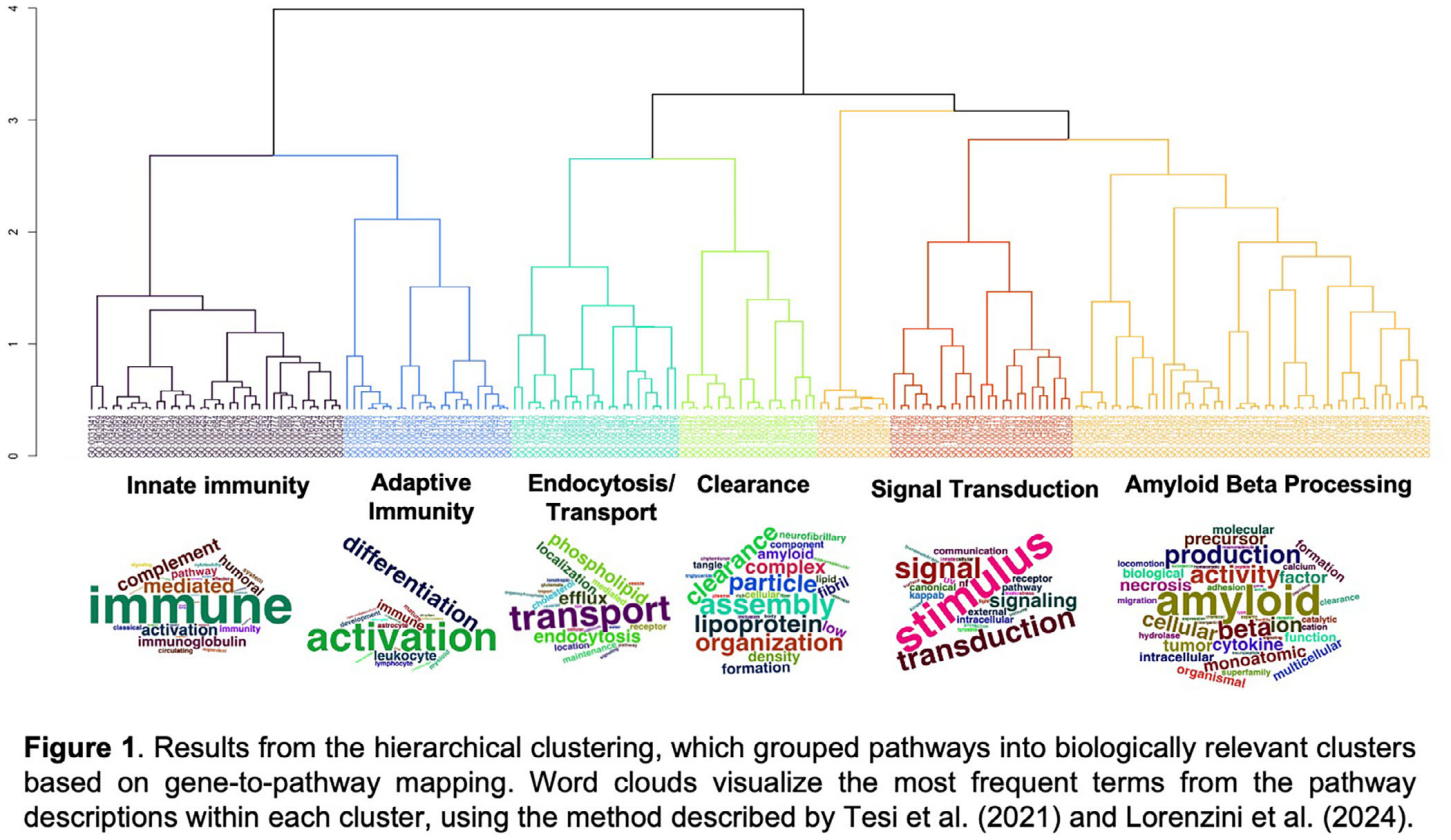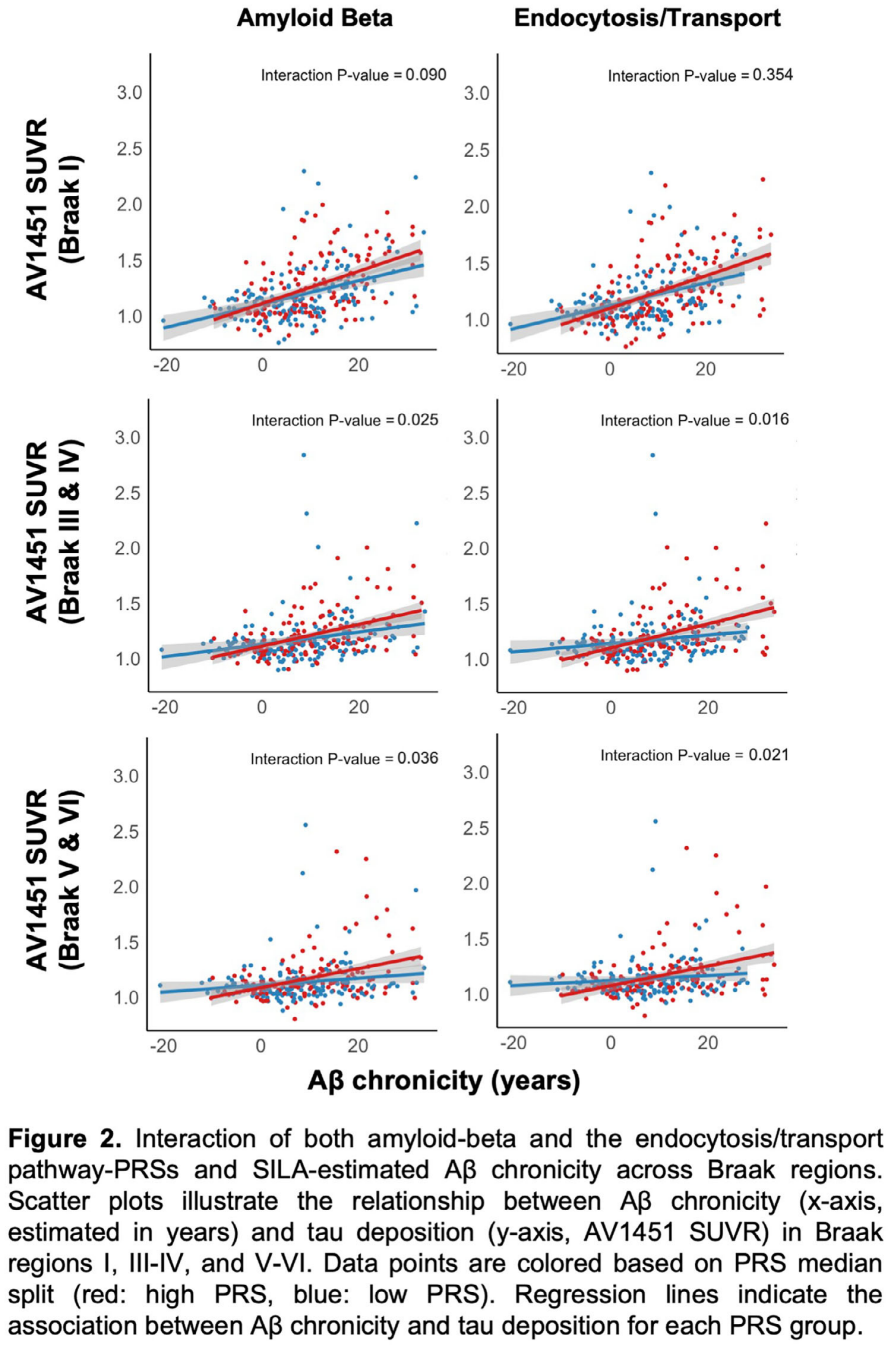# Polygenic risk scores modify the association between amyloid and tau PET accumulation in patients with Alzheimer’s disease

**DOI:** 10.1002/alz70862_110860

**Published:** 2025-12-23

**Authors:** Carolina Valentim, Jannis Denecke, Simon Frerich, Rainer Malik, Nicolai Franzmeier, Michael Ewers

**Affiliations:** ^1^ Institute for Stroke and Dementia Research (ISD), LMU University Hospital, LMU Munich, Munich Germany; ^2^ University of Gothenburg, The Sahlgrenska Academy, Institute of Neuroscience and Physiology, Psychiatry and Neurochemistry, Gothenburg Sweden; ^3^ Munich Cluster for Systems Neurology (SyNergy), Munich, Bavaria Germany; ^4^ German Center for Neurodegenerative Diseases (DZNE), Munich, Bavaria Germany

## Abstract

**Background:**

Genome‐wide association studies (GWAS) have identified a larger number of genetic variants that are associated with increased risk of AD dementia. The biological pathways that underlie the link between SNPs and the development of core AD pathologies remain however unclear. Here, we generated pathway‐specific polygenic risk scores to test their modulating effect on the association between amyloid‐beta (Aβ) chronicity and tau deposition in patients with AD.

**Method:**

We analysed 295 amyloid‐PET‐positive participants from ADNI (mean age 76.5 ± 7.6; 165 CU, 85 MCI, 45 Dementia) with cross‐sectional genetic, cognitive, and tau‐PET (AV1451) and Aβ‐PET (Florbetapir and Florbetaben) data. We identified in a meta‐analysis of recent genome‐wide association studies (GWAS) 388 independent SNPs associated with AD. We computed six pathway‐specific PRS based on gene set enrichment analysis identifying the pathways implicating amyloid beta, immune activation, endocytosis/transport, clearance and signal transduction (Figure 1). Aβ‐chronicity was estimated using the Sampled Iterative Local Approximation (SILA) technique (Betthauser et al., 2022), which infers the temporal trajectory and extent of amyloid accumulation by aligning PET‐derived amyloid burden across longitudinal data points. Robust linear regression (Huber method) models assessed interactions between SILA‐derived Aβ‐chronicity and tau‐PET uptake in Braak‐stage ROIs (I, III‐IV, and V‐VI), controlling APOE‐ε4 status, age, sex and education. Outliers were defined as three standard deviations from the mean.

**Result:**

Significant interactions between Aβ‐chronicity and pathway‐PRS for amyloid beta and endocytosis/transport were observed, with higher genetic risk amplifying the association between longer amyloid exposure and tau accumulation in Braak‐stage ROIs III‐IV & V‐VI (*p* < 0.03). Effects remained significant after the removal of predefined outliers (*p* < 0.02).

**Conclusion:**

Our findings indicate that genetic variations, particularly within the amyloid‐beta and endocytosis/transport pathways, strengthen the association between longer amyloid exposure and increased tau pathology. The significance of the amyloid‐beta pathway suggests a direct genetic contribution to amyloid‐driven tau accumulation, whereas the endocytosis/transport pathway findings support the hypothesis that amyloid‐tau interactions at the synaptic level drive AD progression. These results align with prior evidence on mechanistic links between amyloid and tau pathology in AD.